# Synergy between Vitamin D_3_ and Toll-Like Receptor Agonists Regulates Human Dendritic Cell Response during Maturation

**DOI:** 10.1155/2013/807971

**Published:** 2013-04-07

**Authors:** Anne Brosbøl-Ravnborg, Bettina Bundgaard, Per Höllsberg

**Affiliations:** Department of Biomedicine, Aarhus University, 8000 Aarhus, Denmark

## Abstract

Human dendritic cells (DC) can be differentiated from blood monocytes in the presence of GM-CSF and IL-4 and matured by lipopolysaccharide (LPS). Vitamin D_3_ inhibits the maturation of human DC measured by changes in surface expression of HLA-DR, CD14, CD40, CD80, CD83, and CD86. We here examine the function of vitamin D_3_ during DC maturation. One of the earliest changes to LPS-induced maturation was an increase in CD83 expression. Vitamin D_3_ inhibited the increase in expression of HLA-DR, CD40, CD80, CD83, and CD86 and the decrease in expression of CD14, which was paralleled morphologically by vitamin D_3_-induced inhibition of dendritic cell differentiation. Vitamin D_3_ acted in synergy with the TLR agonists LPS and peptidoglycan (PGN) in inducing IL-6, IL-8, and IL-10, whereas vitamin D_3_ completely inhibited LPS-induced secretion of IL-12. The synergy occurred at concentrations where neither vitamin D_3_ nor the TLR agonists alone induced measurable cytokine secretion. Both LPS and PGN enhanced the level of the vitamin D_3_ receptor (VDR). Taken together, these data demonstrated that vitamin D_3_ and TLR agonists acted in synergy to alter secretion of cytokines from human DC in a direction that may provide an anti-inflammatory environment.

## 1. Introduction

Dendritic cells (DC) are highly specialized, professional antigen-presenting cells (APC) that orchestrate the immune response via integration of a variety of signals [1]. Immunosuppressive and anti-inflammatory compounds like IL-10 [2], 1*α*,25-dihydroxyvitamin D_3_ [3–5], and TGF-*β* [6] induce DC with tolerogenic properties *in vitro*. These DC are generally characterized by an immature or semimature phenotype, with low expression of costimulatory molecules. In addition, tolerogenic DC produce low amounts of proinflammatory cytokines and high amounts of anti-inflammatory cytokines.

Interactions between DC and regulatory T cells (Treg) facilitate the immunosuppression, immature DC induce Treg, and vice versa Treg prepare DC to become immunosuppressive [7, 8].

The biological active form of vitamin D is mediated by the active hormonal form 1*α*,25-dihydroxyvitamin D_3_ (hereafter referred to as vitamin D_3_). Its intracellular transcriptional effects are mediated through binding to the vitamin D receptor (VDR), which subsequently functions as a transcription factor. VDR is constitutively expressed in APC, such as macrophages and DC, and is inducible in activated T lymphocytes [9–11].

Several studies have demonstrated immunosuppressive effects of vitamin D_3_ on the functions of DC. *In vitro* maturation of both human and mouse DC in presence of vitamin D_3_ lead to reduced expression of MHC-II and the costimulatory molecules CD40, CD80, and CD86 resulting in an enhanced production of IL-10 and a reduced secretion of IL-12 [12, 13].

Vitamin D_3_ deficiency has been associated with a higher rate of several diseases, including multiple sclerosis (MS) [14, 15]. Moreover, administration of vitamin D_3_ in the animal model experimental autoimmune encephalitis (EAE) suppressed the development and progression of disease [16], and vitamin D_3_ has also been shown to ameliorate several other models of autoimmune diseases [17, 18].

Infection with Gram-negative bacteria provides lipopolysaccharide (LPS), which contains pathogen-associated molecular patterns (PAMP) that function as a Toll-like receptor (TLR) ligand. LPS is detected by TLR4 and is one of the major components used for inducing maturation of monocyte-derived DC *in vitro* [19]. TLR are expressed by many APC and activate an intracellular signaling pathway that leads to transcriptional activation of proinflammatory genes and innate effector molecules [20]. 

It is feasible that PAMP and TLR ligands interfere with vitamin D_3_ functions in the immune system. This would be consistent with the fact that individuals with severe vitamin D_3_ deficiencies have an increase susceptibility to intracellular infections [21]. A recent study shows that the influence of vitamin D_3_ on TLR4 ligand-induced activation of APC is dependent on the order of VDR and TLR4 engagement [9]. To further study the interplay between TLR agonists and vitamin D_3_, we examined the maturation and cytokine profile of DC differentiated *in vitro* from human peripheral blood monocytes.

## 2. Materials and Methods

### 2.1. Ethical Approval

The study was conducted in accordance with the Ethical Declaration of Helsinki. The project was approved by the local Ethics Committee on Biomedical Research Ethics (j. no20090210). 

### 2.2. Isolation of Peripheral Blood Mononuclear Cells

Human peripheral blood mononuclear cells (PBMC) were isolated from buffy coats (Blood bank, Aarhus University Hospital, Skejby, Denmark) of healthy donors using Ficoll-Paque PLUS (GE Healthcare BioScience AB, Uppsala, Sweden) with density gradient centrifugation according to the manufacturer's procedure. PBMC were cryopreserved in 90% heat-inactivated fetal bovine serum (FBS) (Sigma-Aldrich, Saint Louis, USA) supplemented with 10% dimethyl sulfoxide (DMSO) (Sigma-Aldrich, Saint Louis, MO, USA) at a concentration of 10^7^ cells/mL and stored at −80°C until use.

### 2.3. Isolation of CD14^+^ Monocytes by EasySep Negative Selection

For the isolation of monocytes, PBMC were rapidly thawed and resuspended in phosphate-buffered saline (PBS) supplemented with 2% FBS and 1 mM EDTA. From these cells, monocytes were purified by negative immunomagnetic depletion using the EasySep Human Monocyte Enrichment Kit according to manufacturer's instructions (cat. no 19059, Stemcell Technologies, Grenoble, France). In brief, cells were resuspended at a concentration of 5 × 10^7^ in PBS (without Mg^2+^ and Ca^2+^) + 2% FBS + 1 mM EDTA. First, cells were labeled with EasySep Human Monocyte Enrichment Cocktail for 10 min at 4°C, and then the EasySep D Magnetic Particles for Monocytes were incubated with the cell suspension for 5 min at 4°C. Finally, the suspension was placed into the EasySep Magnet, and the desired negatively selected untouched monocytes were collected. To increase purity, the magnetic isolation procedure was repeated once, and the harvested cells were kept cold. The purity of the untouched monocytes was evaluated by flow cytometry for CD14 expression. Cells were collected and labeled with FITC-conjugated mouse anti-CD14 monoclonal antibody (TÜK4, DAKO Denmark A/S, Glostrup, Denmark). Cell populations were analyzed on a Cytomics FC500 flow cytometer (Beckman-Coulter) and with FlowJo software (Tree Star, Ashland, USA). The purity ranged from 85 to 95% (data not shown). 

### 2.4. Generation of Monocyte-Derived Dendritic Cells

The purified monocytes were washed twice and cultured in 6-well plates (Techno Plastic Products) at a density of 0.5–0.7 × 10^6^ cells/mL in 1.2 mL of RPMI 1640 medium (Gibco, LifeTechnologies, UK) supplemented with 2% normal human serum type AB (NHS AB), 2 mM glutamine, and 20 *μ*g/mL gentamicin (Gibco). The cultures were established in the presence of 100 ng/mL recombinant human GM-CSF (Bayer Healthcare AG, Leverkusen, Germany or Peprotech, London, UK) and 20 ng/mL recombinant human IL-4 (Immunotools GmbH, Friesoythe, Germany). The plates were incubated at 37°C with 5% CO_2_ for six days. On day 3, the cultures were supplemented with 360 *μ*L fresh medium supplemented with 100 ng/mL GM-CSF and 20 ng/mL IL-4. On day 5, 1.56 mL fresh medium supplemented with 100 ng/mL GM-CSF and 20 ng/mL IL-4 was added to the cultures together with 0, 20, or 100 nM of 1*α*,25-dihydroxyvitamin D_3_ (Sigma-Aldrich) as indicated. On day 6, either mock (medium), bacterial LPS (*E. coli *O111:B4, LPS ultrapure cat. no. tlrl-3pelps, InvivoGen, San Diego, USA) in concentrations of 0, 0.01, 0.1, 1, 10, or 100 ng/mL, or PGN (from *S. aureus*, cat. no. tlrl-pgnsa, InvivoGen) in concentrations of 0, 0.1, 0.5, 1, 5, or 10 *μ*g/mL was added during the last 24 hrs of culture. On day 7, all supernatants were collected and frozen at −70°C and all cells were harvested for subsequent phenotypic and functional analyses. 

### 2.5. Morphological Examination

In order to examine the morphological maturation of the cells, viability and morphology were evaluated by light microscopy (Leica Microsystems) of the cells in the 6-well plates at a magnification of ×10 or ×40 prior to harvest.

### 2.6. Flow Cytometric Analyses

Harvested cells were washed twice in PBS and resuspended in 100 *μ*L PBS supplemented with 0.5% bovine albumin serum (BSA) and 0.1% sodium azide for staining. Cells were incubated for 10 min at room temperature with an Fc receptor blocking solution (BioLegend, San Diego, USA). Next, cells were incubated at 4°C for 30 min with the following monoclonal mouse-anti-human antibodies (mAb): anti-HLA-DR FITC (L243, BD Biosciences), anti-CD14 FITC (TÜK4, DAKO), anti-CD40 PE (5C3, BD Biosciences), anti-CD80 PE (MAB104, Beckman Coulter), anti-CD83 PE-Cy5 (HB15e, BD Biosciences), and anti-CD86 PE-Cy5 (2331 FUN-1, BD Biosciences). After staining, cells were washed twice in PBS supplemented with 0.5% BSA and 0.1% sodium azide and resuspended in PBS with 0.99% paraformaldehyde. Flow cytometry was performed on a Cytomics FC-500 flow cytometer (Beckman Coulter), and all subsequent analyses were made in FlowJo software (Tree Star).

### 2.7. Quantification of Cytokines

For cytokine assessment, supernatants from DC cultures were thawed and centrifuged shortly and the content of IL-6, IL-8, IL-10, and IL-12p70 was measured with enzyme-linked immunosorbent assay (ELISA). Concentrations of IL-6, IL-8, IL-10, and IL-12p70 were measured using DuoSet ELISA kits (R&D Systems) according to the manufacturer's instructions. The spectrophotometer Versamax ELISA microplate reader (Molecular Devices, LLC, Sunnyvale, USA) and Softmax Pro software (Molecular Devices) were used to measure and analyze the samples.

### 2.8. Western Blotting Analysis/Cell Lysis and Immunoblotting

Whole-cell extracts were prepared using 1x lysis buffer (Cell Signaling Technology, Beverly, MA, USA) supplemented with 1 mM PMSF, 5 mM NaF, and Complete Mini Protease Inhibitor (Roche Diagnostics, Basel, Switzerland) at a concentration recommended by the manufacturer. Lysates were centrifuged at 2.600 ×g for 5min, followed by 20.000 ×g for 10min, and whole-cell extracts were immediately frozen at −70°C. Proteins were separated in XT Criterion 12* *% gels (BioRad Laboratories Inc, Hercules, CA, USA) using XT MOPS running buffer (Bio-Rad) for 1 h and 30 min at 175 V and subsequently transferred to nitrocellulose membranes for 1 h and 45 min at 300 mA. Detection of VDR was performed using anti-VDR mAb (sc-13133) (Santa Cruz Biotechnology Inc, Dallas, TX, USA) diluted 1 :* * 1000, and GAPDH was detected using anti-GAPDH antibody (Santa Cruz Biotechnology) diluted 1 :* * 2000. The secondary antibodies were horseradish peroxidase-conjugated rabbit anti-mouse or swine anti-rabbit antibody (Dako) diluted 1 : * *2000. All antibodies were diluted in 5* *% skimmed milk in TBS with 0.1* *% Tween-20. Ponceau-S staining was performed as a loading control. Immunoblots were developed using Super Signal West Femto Maximum Sensitivity Substrate (Thermo Scientific, Rockford, IL, USA).

## 3. Results 

### 3.1. LPS Induces Rapid Maturation of DC, Which Is Inhibited by Vitamin D_3_

**Figure 1:**
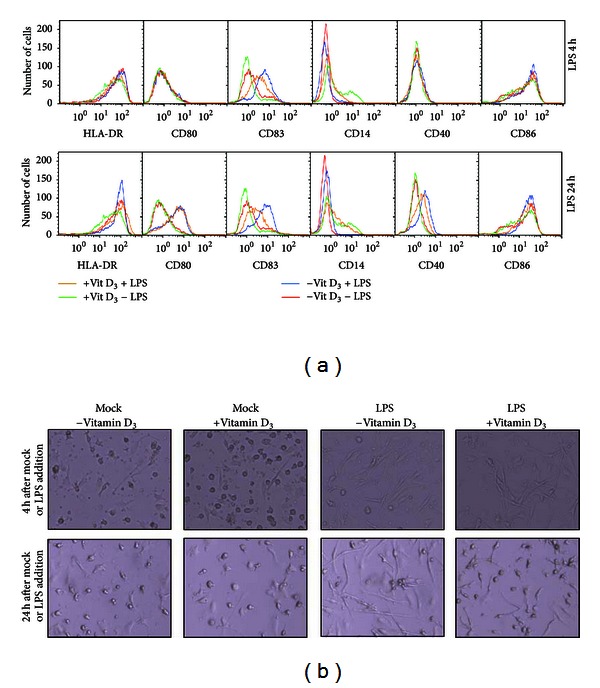
Vitamin D_3_ inhibits LPS-induced DC maturation. (a) DC were differentiated in the presence or absence of vitamin D_3_ and matured with LPS for 4 hrs (upper panel) or 24 hrs (lower panel) prior to flow cytometric analysis. DC were treated without vitamin D_3_ (vit D) and LPS (red curve), without vitamin D_3_ and with LPS (blue curve), with vitamin D_3_ and without LPS (green curve), and with both vitamin D_3_ and LPS (yellow curve) and were stained with fluorescence-conjugated antibodies HLA-DR(FITC), CD80(PE), CD83(PC5), CD14(FITC), CD40(PE), and CD86(PC5). Histograms represent collection of 5,000 events. (b) Morphology of the DC was examined by light microscopy at ×10 magnification at 4 and 24 hrs after mock or LPS treatment. Experiments are representatives of at least two independent experiments.

As expected, vitamin D_3_ added 24 hrs prior to LPS treatment inhibited LPS-induced maturation measured by the expression of surface molecules 24 hrs after the addition of LPS (Figure 1(a)). Conversely, vitamin D_3_ prevented the downregulation of CD14 expression, indicating a block in maturation of immature DC (Figure 1(a)). To further explore the kinetics, cells were examined 4 hrs after addition of LPS. In the absence of vitamin D_3_, DC had already upregulated CD83 and downregulated CD14 after 4 hrs of LPS treatment, whereas only minor or no changes were observed for HLA-DR, CD80, CD86, and CD40. Thus, upregulation of CD83 appears to be an early event during DC maturation. The presence of vitamin D_3_ partially inhibited upregulation of CD83 almost to the same degree as observed after 24 hrs.

The early induction of DC maturation was morphologically visible as dendritic formations as early as 4 hrs after the addition of LPS, but more pronounced after 24 hrs. The presence of vitamin D_3_ inhibited the outgrowth of these processes (Figure 1(b)).

### 3.2. Vitamin D_3_ Is Necessary for LPS-Induced Secretion of IL-6, IL-8, and IL-10

**Figure 2:**
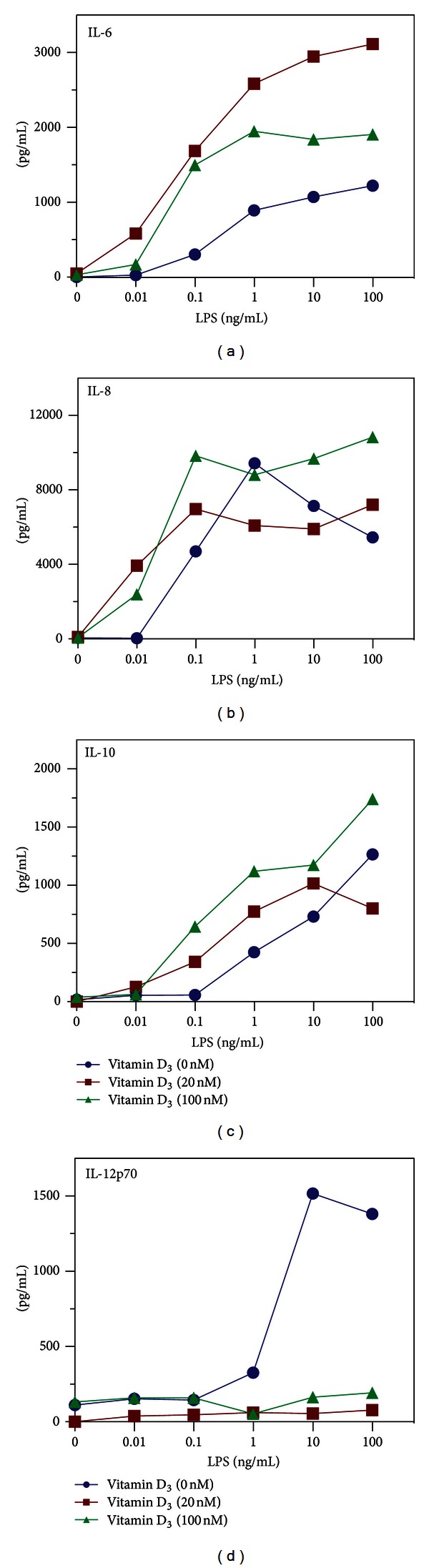
Vitamin D_3_ is necessary for LPS-induced secretion of IL-6, IL-8, and IL-10. DC were stimulated with 0, 20, or 100 nM of vitamin D_3_ (vitamin D) and 10-fold dilutions of LPS (from 100 to 0.01 ng/mL). Cell-culture supernatants were assessed for IL-6 (a), IL-8 (b), IL-10 (c), and IL-12 (d) by ELISA. Experiment is representative of at least two independent experiments.

The presence of vitamin D_3_ inhibited the maturation of differentiated DC. To examine whether vitamin D_3_ influenced the cytokine secretion induced by LPS, differentiated DC were treated with various concentrations of LPS and monitored for the secretion of IL-6, IL-8, and IL-10 in the absence or presence of either 20 or 100 nM vitamin D_3_. These concentrations of vitamin D_3_ did not induce cytokine secretion from DC. Although LPS induced a dose-dependent secretion of IL-6, IL-8, and IL-10, the presence of vitamin D_3_ was able to further enhance this secretion (Figures 2(a)–2(c)). Importantly, at low doses of LPS, cytokines were only induced in the presence of vitamin D_3_, despite the fact that neither LPS alone nor vitamin D_3_ alone induced measurable cytokines. These data demonstrated that vitamin D_3_ acted in synergy with LPS to induce the secretion of IL-6, IL-8, and IL-10.

### 3.3. Vitamin D_3_ Inhibits LPS-Induced IL-12 Secretion

Since vitamin D_3_ is thought to inhibit the proinflammatory response, we next examined whether IL-12, a cytokine that promotes a Th1-like response, was affected by vitamin D_3_. The presence of LPS at concentrations above 1 ng/mL induced high levels of IL-12p70 (Figure 2(d)). In the presence of either 20 or 100 nM vitamin D_3_, the detection of LPS-induced IL-12p70 was completely abolished. This demonstrated that vitamin D_3_ is a potent inhibitor of IL-12. 

### 3.4. Vitamin D_3_ Is Necessary for PGN-Induced Secretion of IL-6, IL-8, and IL-10

**Figure 3:**
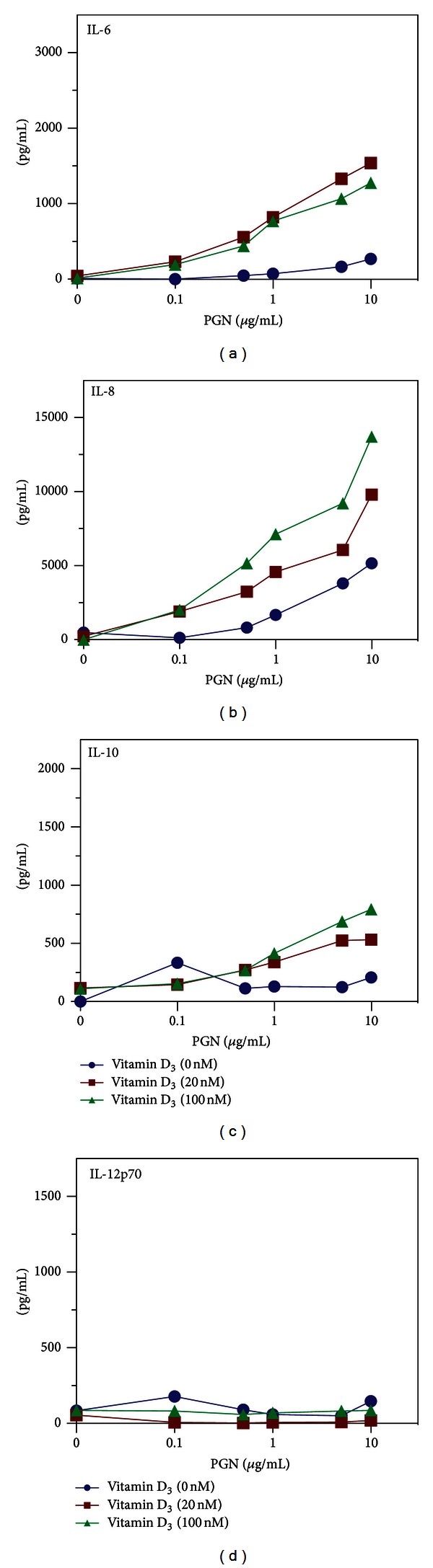
Vitamin D_3_ is necessary for PGN-induced secretion of IL-6, IL-8, and IL-10. DC were stimulated with 0, 20 or 100 nM of vitamin D_3_ and dilutions of PGN (from 0.1 to 10 *μ*g/ml). Cell-culture supernatants were assessed for IL-6 (a), IL-8 (b), IL-10 (c), and IL-12 (d) by ELISA. Experiment is representative of at least two independent experiments.

To test whether LPS was the only TLR ligand able to induce cytokines and act in synergy with vitamin D_3_, differentiated DC were treated with the TLR2 agonist PGN. PGN was able to induce high levels of IL-8 and low levels of IL-6 and IL-10 at high concentrations of PGN (Figures 3(a)–3(c)). However, in the presence of vitamin D_3_, PGN-induced cytokine secretion from differentiated DC was synergistically increased for IL-6, IL-8, and IL-10 (Figures 3(a)–3(c)). In contrast to LPS, PGN did not induce IL-12 (Figure 3(d)). This indicated that PGN, similar to LPS, induced cytokine secretion from DC in synergy with vitamin D_3_.

### 3.5. LPS and PGN Enhance Expression of the Vitamin D Receptor

**Figure 4:**
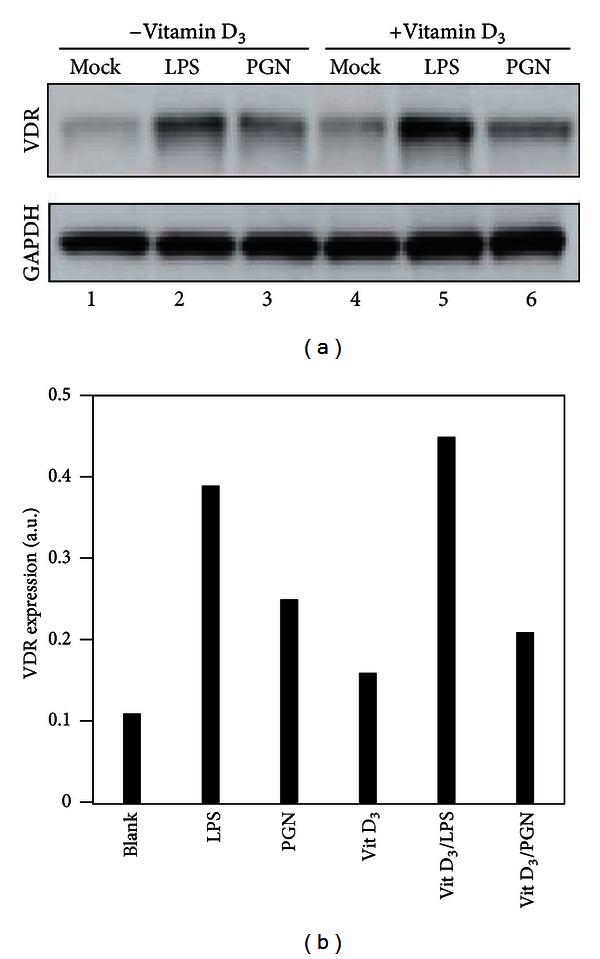
LPS and PGN act in synergy with vitamin D_3_ to enhance expression of the vitamin D receptor. (a) The levels of VDR protein in DC were measured by the western blotting of whole-cell lysates from DC cultures stimulated with either mock, 10 ng/mL LPS, or 10 *μ*g/mL PGN in the acellsbsence or presence of 100 nM vitamin D_3_. GAPDH was used as a loading control. (b) The band intensity in (a) was quantified and normalized to GAPDH as a VDR/GAPDH ratio (VDR expression).

Vitamin D_3_ exerts its function after binding to an intracellular receptor, VDR. To examine whether LPS could increase the sensitivity of DC for vitamin D_3_, the protein level of VDR was measured by western blotting. In the absence of vitamin D_3_ or TLR agonists, a low level of VDR was detectable (Figure 4). However, the presence of LPS or PGN increased the level of VDR, which was not further affected by the presence of vitamin D_3_ (Figure 4). This indicated that LPS or PGN was able to enhance the sensitivity of DC for vitamin D_3_ by increasing the expression of its receptor.

## 4. Discussion

The generation of Treg is in part controlled by the maturation of DC. In mice, treatment with vitamin D_3_ induces a regulatory T-cell profile with increased expression of IL-10, TGF-*β*, FoxP3, and CTLA-4 and a significant reduction of IL-12p70, IL-23p19, IL-6, and IL-17 [22]. We also observed that vitamin D_3_ promoted a regulatory profile, promoting IL-10 and inhibiting IL-12p70. Surprisingly, we found that in synergy with LPS or PGN, vitamin D_3_ increased IL-6 in cultures of differentiated DC. Whether the detection of IL-6 indicates a pro- or an anti-inflammatory profile is, however, not clear. IL-6 may be associated with a soluble form of the IL-6 receptor and this complex may mediate proinflammatory reactions through a process known as transsignaling [23]. Blockade of IL-6 transsignaling completely protected gp130^F/F^ knock-in mutant mice from LPS hypersensitivity, suggesting cross-talk between JAK/STAT and TLR pathways [24]. On the other hand, the classical binding of IL-6 to gp130/IL-6 receptor complexes on the cell surface may promote anti-inflammatory/regenerative reactions [23]. Thus, further investigations may elucidate which one of these scenarios is supported by vitamin D_3_.

Importantly, we found that, at very low levels of LPS, vitamin D_3_ promoted the secretion of IL-8 in synergy with LPS. That is, neither the concentration of 0.01 ng/mL of LPS nor the presence of vitamin D_3_ alone was able to induce this cytokine. However, when added together, vitamin D_3_ and LPS acted in synergy and induced in the order of 2–4 ng of IL-8. IL-8 is a major chemoattractant for the recruitment of polymorphonuclear leukocytes that serve as part of the first line of defense against intruding bacteria. This emphasizes that besides its effect on the adaptive immune system, vitamin D_3_ also has important functions for the innate immune response.

IL-8 has previously been shown to be induced by the transformed cell line THP-1 in the presence of vitamin D_3_ and agonists of TLR2, TLR3, TLR4, NOD1, and NOD2 [25]. However, other studies have reported that vitamin D_3_ inhibited IL-8 production in response to IL-1*α* in human peripheral blood mononuclear cells, keratinocytes, and fibroblasts [26, 27], although TLR and IL-1R are both signals through MyD88. Inhibition of IL-8 by vitamin D_3_ has also been observed in primary cultures of human periodontal ligament cells stimulated with *P. gingivalis*, a Gram-negative bacteria containing LPS [28]. Thus, the cell type, the presence of other cytokines, and potentially virulence factors from microorganisms may all be important for modulating the control exerted by vitamin D_3_ on IL-8 secretion.

Although LPS is frequently used to mature monocyte-derived human DC, other TLR agonist may serve the same functions. LPS is a component of the outer membrane of the Gram-negative bacterial cell wall, whereas Gram-positive bacteria are characterized by a cell wall containing PGN, a ligand for TLR2. In agreement with the observed functions of LPS, PGN also acted in synergy with vitamin D_3_ for the secretion of IL-6, IL-8, and IL-10 but was unable to induce IL-12p70 as did LPS. This indicates that PGN from Gram-positive bacteria may also have the ability to prime the response towards a regulatory T-cell profile and that PGN also induces chemoattractants for recruiting polymorphonuclear leukocytes.

The level of VDR in part determines the sensitivity of DC for vitamin D_3_. Liu et al. found that the *M. tuberculosis*-derived lipopeptide, which is a TLR2/1 ligand, induced the expression of VDR. In addition, they observed that vitamin D_3_ upregulated the antimicrobial peptide cathelicidin, which is important for the killing of intracellular bacteria [19]. However, since most bacteria are extracellular, we examined the general Gram-negative and Gram-positive TLR ligands: LPS (TLR4) and PGN (TLR2). We found that these TLR ligands were also able to increase the level of VDR. The shifting by vitamin D_3_ of the immune response towards a regulatory profile as opposed to a proinflammatory response may be of benefit for the invading microorganisms. In this view, the upregulation of VDR by both LPS and PGN may further enhance the effect of vitamin D_3_ and thus further prevent the antimicrobial defense. The inhibition of IL-8 induction by *P. gingivalis* [28], as mentioned previously, further indicates that the microorganism may have evolved mechanisms to avoid part of these host innate responses. Whether this is a general feature remains to be investigated. Conversely, we speculate that these effects of vitamin D_3_ may also prevent unnecessary inflammatory reactions towards commensal microorganisms present in the gut. The shifting towards a regulatory profile by vitamin D_3_ may also be of benefit during graft-versus-host disease (GVHD) [29]. However, whether the anti-inflammatory actions of vitamin D_3_ can be enhanced in an *in vivo* setting by the addition of low levels of TLR agonists remains to be examined. 

## 5. Conclusions

In conclusion, human DC maturation is inhibited by vitamin D_3_. LPS and PGN (TLR4 and TLR2 agonists) increase the level of VDR and act in synergy with vitamin D_3_ for induction of IL-6, IL-8, and IL-10, whereas LPS induction of IL-12 is inhibited by vitamin D_3_. PGN does not induce measurable IL-12. Thus, vitamin D_3_ synergizes with TLR agonists in modulating human DC cytokine secretion during maturation. This may generate an anti-inflammatory environment that favors the induction of regulatory cells.
